# Hospital Sunday and the Queen's Commemoration

**Published:** 1897-06-26

**Authors:** 


### The Institutional Workshop.

### HOSPITAL SUNDAY AND THE QUEEN'S COMMEMORATION.

It may be a coincidence merely, but we feel it may properly be regarded as providential, in the special circumstances, that, although the new rector of St. Margaret's, Westminster, had arranged not to have a collection on behalf of the hospitals last Sunday but to devote the offertories to an object confined entirely to the Organ Fund of the church, which has one of the wealthiest congregations in London, the debt being discharged by two members of the House of Commons, all the contributions of the congregation were appropriated to the Metropolitan Hospital Sunday Fund. It seems to us that the very few London clergy who, in face of the arrangement made in February last by the present Archbishop of Canterbury, then Bishop of London, for the collection on behalf of the hospitals in 1897, devoted the offerings to any other object than the Metropolitan Hospital Sunday Fund, showed a lack of patriotism and a want of appreciation of the spirit which has moved the hearts of the whole of the people of these realms, and especially of the metropolis of the Empire, in connection with the Queen's Commemoration. It is remarkable that there should be a single clergyman or minister of religion in the whole metropolis whose heart was not warmed and whose imagination was not fired by the thought that he, in his divine office, should have had the privilege on Accession Day, of all days in the year, to plead the cause of the suffering and the sick. If there is one feature more prominent than another in the reign of Queen Victoria it is the noble example set by Her Majesty and all the members of her family in supporting with their influence and by personal service the cause of the voluntary charities, and especially of the hospitals. Any minister of God who had the privilege of preaching from a metropolitan pulpit on June 20th, 1897, must have devoted even more care than usual to the preparation of his address; and he must have felt that any remarks he might make would fail to be worthy not only of the great national event and the particular day, but unworthy of the Christianity of which he was a teacher and upholder, should he in the special circumstances fail to plead for the weak, the suffering and the sick, who owe more to Christian teachings as the source of comfort in their hour of trouble than to anything whatever. To talk about dragging in the hospitals, as one gentleman is reported to have done, is to talk nonsense. Christianity is not a dead faith; it has a tender regard for and an ever warm inclination towards the souls and bodies of men. It is its purpose to minister to the sick, to comfort the broken-hearted, and to uphold the fallen. From whatever aspect the preacher might determine to consider the progress of the British Empire and of the people of these realms during the last sixty years, he could not, without violence to his profession and faith, omit to mention the surprising development in the means now provided for the alleviation of suffering, for the care of the sick, and for the treatment of all who, from whatever cause, find themselves in the position of the man who fell among thieves. We rejoice, therefore, to know that the clergy of the capital of the British Empire, the greatest city on the face of the earth, determined, with a few insignificant exceptions, not to turn their back upon the parable of the good Samaritan and the teaching it embodies, when dealing with the sixtieth anniversary of the greatest reign in English history.

Had they done so they must have lamentably failed in their duty, and in practice must have disowned the faith which it is their privilege to teach and uphold. The congregations were, we have reason to know, anxious by their contributions to show the gratitude which they feel for the mercies they have received under Queen Victoria, as well as their loyalty and affection for her person. We shall not be surprised, therefore, to learn, as we hope to be able to report, that the total sum received from the collections in the churches and chapels of London on Hospital Sunday, 1897, have far exceeded the amount which has ever before been given through this channel for the relief of the suffering and the sick.

Apart from the immediate object of the services throughout London, not only those at St. Paul's Cathedral and Westminster Abbey, but those in the humblest places of worship, were marked by a spirit of fervent devotion and heartfelt thankfulness, typifying unity in its noblest and best aspect, and this feature of unity must ever be memorable in our history. No one who was present at these services could have failed to be impressed by the conduct of the congregation, and by the hearty and united way in which the people present entered into the spirit of the occasion and the day. We are glad, however, to note the *Times*' expression of regret at certain members of the congregation at the morning service at St. Paul's who so far forgot themselves as to stand on their chairs in order to obtain a better view of their Royal Highnesses as they walked down the nave on leaving the Cathedral. At Westminster Abbey it was noted, too, that a very small minority of individuals interrupted the service by leaving after the Dean had ascended the pulpit, and during the progress of his address. We think it to be due to the place and the occasion that for the future, whenever special services take place like those of last Sunday, the authorities of St. Paul's and the Abbey should clearly intimate that anyone to whom a ticket is issued is expected to remain in his place until the end of the service, and that any departure from this rule will entail the taking down of the name and address of the offender, as the equivalent of a refusal to issue tickets to him on any similar occasion. The conduct we refer to—and we were sorry to see among the offenders a member of the Cabinet and his family—not only shows irreverence, but is so foreign to the spirit of the occasion, and so interferes with the act of worship, that apart from the bad manners it displays it demands the application of an effectual remedy. The pained expression noticeable on the faces of the vast majority of the congregation present should have served as a check, but unfortunately this was not only disregarded, but some of those who left were not even careful to leave quietly and unobtrusively. We should not have ventured to refer to the matter in this place did not our experience make us feel that the habit is a growing one amongst a certain class of people who ought to know better, but who seem incapable of exhibiting in public on these occasions any other feeling than one of selfishness combined with a disregard to the place and what is due not only to the occasion, but to those assembled and to themselves also as presumably people of education and good manners.

The services at St. Paul's, at Westminster Abbey, and at the Great Synagogue, and no doubt in many other places of worship, were probably the most remarkable, interesting, and memorable which have ever taken place. The beauty of the music, the evident determination of readers and preachers to be heard and understood, and the marked absorption and devotion of the enormous congregations proved to demonstration how all-pervading was the spirit of worship, love and thankfulness, and how absolutely the great wife, mother, friend, and Queen had won her people's hearts.

How it would have rejoiced the heart of Canon Miller, the founder of Hospital Sunday, to have seen this day, to have been privileged to take part in the services, and to find that his idea of uniting the churches in the cause of the sick has so fructified during the last half of the Victorian Era that what was once thought a mere ideal has become in practice not only a reality, but a binding force for good, which can never fail to bear fruit to the glory of God and the ennoblement of humanity.

Of course this article was necessarily written before the results of the collections on Hospital Sunday were known. It is therefore our great pleasure to be able to state, on the authority of the Chief Rabbi (the Rev. Dr. Adler), one of the warmest and most valued friends of the Hospital Sunday Fund, that his usual collection at the Great Synagogue on Hospital Sunday has been about £250, but that this year he has succeeded in raising no less a sum than £550. In regard to many of the other churches the official returns have not yet been received, probably in consequence of the holidays.

